# Supplementary material for: MetaRibo-Seq measures translation in microbiomes
Source: Nat Commun. 2020 Jun 29;11:3268. doi: 10.1038/s41467-020-17081-z (PMC7324362; doi:10.1038/s41467-020-17081-z)
Supplement: Supplementary file 10 — Supplementary Data 7 [file 41467_2020_17081_MOESM10_ESM.zip › File2/Confidence_High_Taxonomy/291483_out.krona.html]

Javascript must be enabled to view this page.

members
magnitude
magnitudeUnassigned
count
unassigned
taxon
rank

291483\_out

3

superkingdom
3
2

phylum
3
976

200643
3
class

3
order
171549

family
3
171552

3
genus
838


SRS015794\_contig\_number\_4487
2292365
1
species


SRS049959\_contig\_number\_38248
165179
1
species


SRS012849\_contig\_number\_13945
2292054
1
species
